# Treatment of Pancreatic and Periampullary Cancers at a Community Hospital: Successful Application of Tertiary Care Treatment Standards

**DOI:** 10.1155/2011/936516

**Published:** 2011-12-19

**Authors:** Robert C. Moesinger, Jan W. Davis, Britani Hill, W. Cory Johnston, Carl Gray, Harold Johnson, Leslye Ingersoll, Gary Whipple, Mark Reilly, Robert Harris, Vincent Hansen

**Affiliations:** ^1^Department of Surgery, McKay-Dee Hospital Center, Ogden Regional Medical Center, Ogden, UT 84405, USA; ^2^Department of Surgery, Medical Oncology, Ogden Regional Medical Center, Ogden, UT 84405, USA; ^3^Department of Surgery, University of Utah, Salt Lake City, UT 84132, USA; ^4^Department of Medical Oncology, McKay-Dee Hospital Center, Ogden Regional Medical Center, Ogden, UT 84405, USA; ^5^Department of Radiation Oncology, McKay-Dee Hospital Center, Ogden, UT 84403, USA; ^6^Department of Radiation Oncology, Ogden Regional Medical Center, Ogden, UT 84405, USA; ^7^Department of Medical Oncology, Chairman of Cancer Committee, McKay-Dee Hospital Center, Ogden, UT 84405, USA

## Abstract

*Background*. The treatment of pancreatic cancer and other periampullary neoplasms is complex and challenging. Major high-volume cancer centers can provide excellent multidisciplinary care of these patients but almost two-thirds of pancreatic cancer patients are treated at low volume centers. There is very little published data from low volume community cancer programs in regards to the treatment of periampullary cancer. In this study, a review of comprehensive periampullary cancer care at two low volume hospitals with comparison to national standards is presented. *Methods*. This is a retrospective review of 70 consecutive patients with periampullary neoplasms who underwent surgery over a 5-year period (2006–2010) at two community hospitals. *Results*. There were 51 successful resections of 70 explorations (73%) including 34 Whipple procedures. Mortality rate was 2.9%. Comparison of these patients to national standards was made in terms of operative mortality, resectability rate, administration of adjuvant therapy, clinical trial participation and overall survival. The results in these patients were comparable to national standards. *Conclusions*. With adequate commitment of resources and experienced surgical and oncologic practitioners, community cancer centers can meet national tertiary care standards in terms of pancreatic and periampullary cancer care.

## 1. Introduction

Pancreatic adenocarcinoma is the 4th leading cause of cancer death in the United States. The National Cancer Institute estimated that there were 43,140 new cases of pancreatic cancer in the United States in 2010 and that pancreatic cancer was responsible for 36,800 deaths [1]. Other periampullary malignancies requiring similar treatment are less common and include distal common bile duct cancer, duodenal cancer, and ampullary cancer. Standard of care treatment of these cancers and especially pancreatic cancer requires a multidisciplinary approach and many components of treatment require special expertise, that is, biliary-pancreatic surgery, endoscopy, and radiology.

High-volume tertiary care centers and cancer institutions have greatly advanced the care of pancreatic cancer patients over the last 4 decades, and there are a number of studies indicating the superior surgical results [2–5] and possibly long-term survival [6] for patients cared for at such facilities. Nevertheless, it is impossible for all pancreatic cancer patients to be cared for at regional cancer centers and a significant number of patients receive their pancreatic cancer care at community institutions [7]. Some studies note that volume is an imperfect surrogate for quality at best [8]. Low- and medium-volume centers can definitely provide excellent complex cancer care given adequate resources and experienced practitioners [9]. Many patients and families desire to have their cancer care at local facilities for various reasons [10]. These include the burden of travel and lodging at out-of-town institutions as opposed to the familiarity and easier access to local physicians and hospitals. Consequently, it is desirable to have standard of care pancreatic cancer treatment at community medical facilities where practical.

This paper describes the introduction of pancreatic surgery at two medical centers in Ogden, Weber County, Utah—McKay-Dee Hospital Center (MKD) and Ogden Regional Medical Center (ORMC), and the results of the subsequent multidisciplinary care of pancreatic and other periampullary cancer patients. Prior to the introduction of pancreatic surgery in Weber County in August, 2006, the vast majority of pancreatic cancer patients in this area were referred to Salt Lake City, some 40 miles away.

## 2. Materials

We retrospectively reviewed all patients (70) who underwent resection of pancreatic adenocarcinoma, other pancreatic and periampullary neoplasms or palliative surgery for unresectable periampullary cancer over a 4.5-year period beginning in August 2006 at MKD and beginning in January 2008 at ORMC. It was at this time that one of the authors with experience in pancreatic surgery (RCM) joined the staffs of the two hospitals in Ogden, UT. Our retrospective review includes patient pathologies, surgical results, adjuvant therapies, and survival. Additionally, patient survival, through December 2010, for pancreatic cancer was compared stage for stage to the results documented in the National Cancer Data Base (NCDB) of the American College of Surgeons Commission on Cancer [11]. Results and basic descriptive statistics are presented.

## 3. Geographic Area Served

Utah, the 13th largest state in the United States, is a large (84, 904 square miles) [12], mostly rural state with a single “strip” of urban demographics covering 4 counties (Weber, Davis, Salt Lake and Utah) in northern Utah with Salt Lake City at the center of this urban “strip.” These 4 counties, known as the “Wasatch Front” as they run between the Wasatch Mountains and the Great Salt Lake, contain 76% of Utah's population. (2,785,000—2009 estimate) [13].

**Figure 1:**
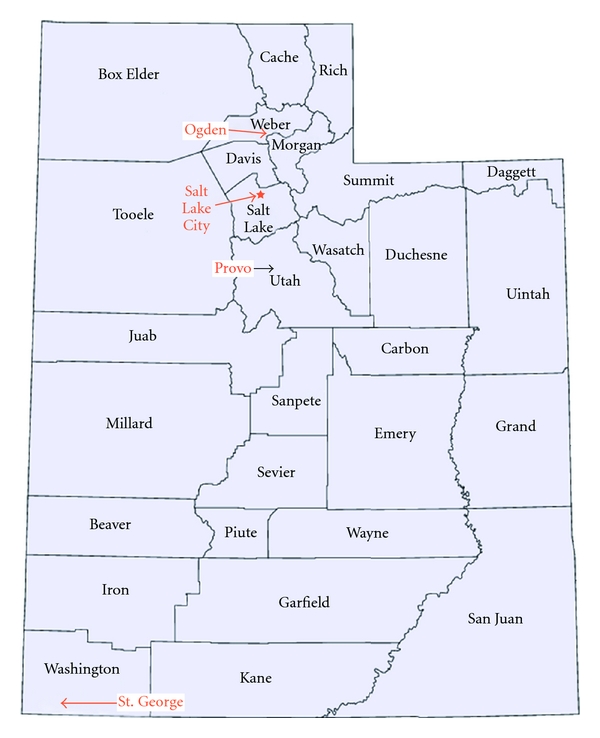
State of Utah, with counties and select cities indicated.

Pancreatic cancer in Utah (2003–2007 data) has an incidence of 9.7 per 100,000 with an annual mortality rate of 8.9 per 100,000. This translates to about 250 cases per year and a total of 220 deaths per year in Utah [14]. Prior to 2006, radiation and chemotherapy for pancreatic cancer were delivered at a number of medical centers around the state, but almost all pancreatic cancer surgical care, particularly Whipple procedures, was delivered in Salt Lake City, mostly at either the University of Utah Medical Center or LDS Hospital. A handful of pancreatic cancer surgeries are also done at other hospitals in Salt Lake City as well as in Provo and St. George. Clearly another center for the delivery of comprehensive pancreatic cancer care was highly desirable, see Figure 1.

## 4. McKay-Dee Hospital Center

McKay-Dee Hospital Center, owned by Intermountain Healthcare, is a 317-bed community hospital which provides comprehensive medical and surgical care [15]. Located in Ogden, Utah (2006 population 78,100 [13]), it serves patients primarily in Weber, Morgan, and northern Davis Counties (2009 populations 232,000, 8,900, and 301,000, resp.) [13] in northern Utah. However, it serves as a tertiary referral center and draws patients from a vast geographic area including all of northern Utah, north of Salt Lake City, as well as southwestern Wyoming and southeastern Idaho. It has an American College of Surgeons Committee on Cancer accredited cancer program.

## 5. Ogden Regional Medical Center

Ogden Regional Medical Center [16], owned by MountainStar, is a 238-bed community hospital which provides comprehensive medical and surgical care to the same region as McKay-Dee Hospital. They are, in fact, 2.5 miles apart, and they are the only hospitals in Weber County.

## 6. Patient Management

Both facilities have a well-functioning cancer committee for presentation and discussion of complex patients. Most of the pancreatic cancer patients are seen in a multidisciplinary GI cancer clinic to facilitate patient care and treatment planning. Biliary endoscopy, diagnostic and interventional GI radiology, state-of-the-art surgical care, and full-time intensivists are available at both facilities.

Radiation therapy at both facilities is based on state of the art treatment guidelines. Three-dimensional conformal or IMRT radiation therapy techniques are utilized, with no fewer than four to seven treatment angles. Dose to the prescribed volume is generally 4500 cGy, followed by a conedown boost to the tumor bed or unresected tumor and margin for an additional 540 to 900 cGy. Dose volume histograms are used to analyze dose to the multiple surrounding normal structures, including the liver, kidneys, small bowel, and spinal cord. Multiple plans are tried, with or without couch angles, to minimize dose to normal structures.

Administration of chemotherapy generally consists of intravenous gemcitabine (Gemzar) 1000 mg on days 1, 8, 15, 21, and 28. Then after concurrent radiation with continuous infusion 5-Fluorouracil (5-FU) 250 mg/m^2^, intravenous gemcitabine is continued 1000 mg on days 1, 8, 15 every four weeks for three to six months.

## 7. Results

**Figure 2:**
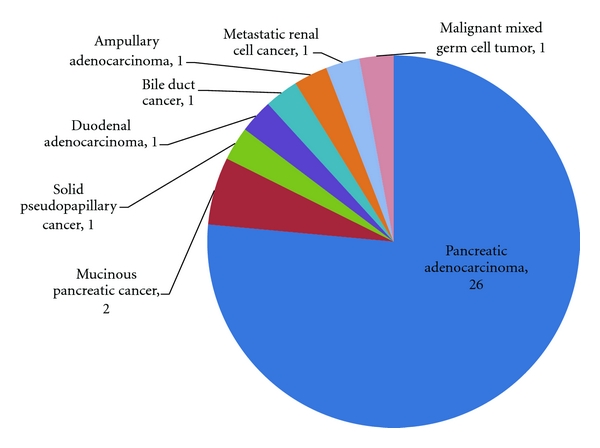
Resected Cancers (*n* = 34).

**Figure 3:**
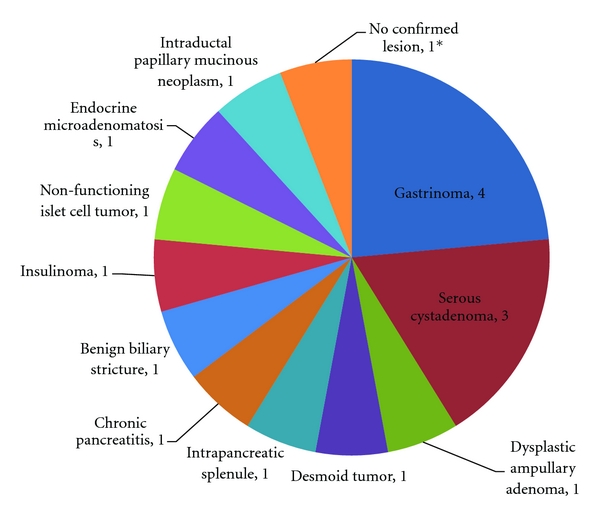
Benign Lesions (*n* = 17). *This patient had a mass in the tail of the pancreas on CT with an elevated CA 19-9 which resolved after distal pancreatectomy, but no clear adenocarcinoma could be confirmed on pathology.

**Figure 4:**
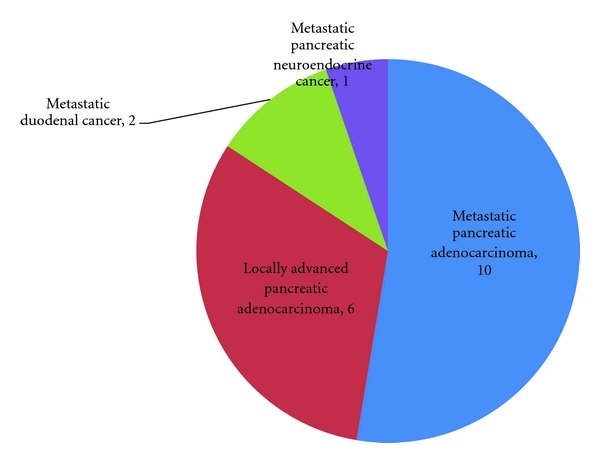
Unresectable Cancers (*n* = 19).

Over the 4.5 years of this review, a total of 70 pancreatic resections were performed or attempted by a single surgeon (RCM). These included 34 resected cancers, 17 resections for benign disease, and 19 palliative procedures. The most common pathology was pancreatic adenocarcinoma with 28 resected adenocarcinomas and 16 unresectable adenocarcinomas for a total of 44 surgical pancreatic cancer patients. The unresectable adenocarcinomas included 10 metastatic lesions and 6 locally advanced lesions with extensive vascular involvement. The pathologies for the resected cancers, the benign lesions, and the palliative procedures are summarized in Figures 2, 3, and 4, respectively.

 The procedures performed included 26 pylorus-preserving Whipple procedures, 7 classic Whipples, 12 distal pancreatectomies, 1 total pancreatectomy, 1 middle-segment pancreatectomy, and 4 enucleations. The 19 palliative procedures included 11 gastrojejunostomies, 6 combined gastrojejunostomies and hepaticojejunostomies, 1 laparoscopic liver biopsy, and 1 pancreas-sparing partial duodenectomy. (This was for a bleeding duodenal cancer in a patient with metastatic disease.) An alcohol neurolysis of the splanchnic nerves was usually performed with the open palliative procedures. There were two 30-day mortalities (2.9%). Among the patients with pancreatic adenocarcinoma (44), there was one operative mortality (2.3%). Among patients who had Whipple procedures (34), there was one operative mortality (2.9%), and the mortality rate was 2% (1 of 51) for all pancreatic resections.

Of the 28 resected pancreatic adenocarcinomas, 24 patients (86%) completed a full course of radiation therapy, most of which also had concurrent continuous infusion 5-FU. Of these, one was in a neoadjuvant setting prior to resection of a pancreatic body lesion. Of the 4 patients who did not receive a full course of radiation therapy, one declined all adjuvant therapy; one stopped therapy due to side effects; one was on the RTOG 0848 [17] protocol and was randomized to no radiation therapy, and one developed metastasis prior to planned radiotherapy and therefore did not proceed. Of the 6 patients with locally advanced (not metastatic) disease, one had a full course of chemoradiation in the neoadjuvant setting, but remained unresectable at exploration. Two had a full course of chemoradiation after exploration. Two had incomplete radiotherapy due to intolerance, and one was lost to followup.

In terms of adjuvant chemotherapy, 26 (93%) of the patients with resected adenocarcinomas received gemcitabine- (Gemzar-) based chemotherapy. The other two patients declined chemotherapy. Two of the 26 received Gemzar in the neoadjuvant setting. Four of the 26 received Gemzar in combination with other agents including Xeloda, Avastin, Tarceva, and Erlotinib. All of these patients were on study protocols. Of the unresected patients, nine patients received Gemzar chemotherapy, four of which also received other agents (Xeloda, Oxaliplatin, Erlotinib, FOLFOX). Two patients declined chemotherapy. Two received 5-FU with radiation but no further chemotherapy. Three were lost to follow up. Two of the patients received neoadjuvant chemotherapy (one with radiation as well) but remained unresectable at exploration.

Of the 44 patients with pancreatic adenocarcinoma, six (14%) participated in a clinical trial, five at McKay-Dee Hospital, and one at the University of Utah. These clinical trials included RTOG 0848 [17], E2204 [18], and SWOG 0727 [19].

## 8. Survival

The median followup for all 44 pancreatic cancer patients is 9 months. For those patients still living (25), the median followup is 10 months. For the 10 patients with metastatic (Stage IV) pancreatic cancer, 7 are dead with an average survival of 6.6 months. For the six patients with locally advanced pancreatic cancer (Stage III), three are dead with an average survival of 5 months. The six remaining unresected patients remain alive 1–10 months after their surgery. An additional patient who presented with Stage III disease underwent neoadjuvant chemo- and radiation therapy followed by a distal pancreatectomy and splenectomy and is alive at 27 months.

 For the 28 resected pancreatic cancers, the median survival has not yet been reached with 19 still living. The average survival for those still living is 18 months (range 4–49). The average survival for those patients who have died is 17 months (range 4–32). Actual 1-, 2-, and 3-year survival is demonstrated in Table 1. Overall, 86% of patients (18 of 21) who are at least one year postsurgery have survived one year, and 62% of patients (8 of 13) who are at least two years postsurgery have survived two years. Comparison to National Cancer Data Base results is illustrative: 1-year, 2-year and 3-year survivals for Stage I disease are 47%, 28%, and 21%, respectively. For Stage II disease the 1-year, 2-year and 3-year survivals are 50%, 26%, and 16%, respectively [11]. 

## 9. Discussion

This review demonstrates the results of the introduction of pancreatic surgery and multidisciplinary pancreatic cancer care at two community hospitals over a 4.5-year period. This introduction proceeded smoothly in large part due to excellent oncologic and gastroenterologic resources already in place. Very active cancer committees and administrative support facilitated this process.

Published series of pancreatic cancer experience from low-volume community centers are few [9, 20, 21] despite the fact that low-volume centers take care of the majority of these patients—61%, based on the Chang review of Nationwide Inpatient Sample data [7]. It is clear that quality and outcomes can vary considerably at low volume centers. Nevertheless, our data as well as others support the idea that a community facility with adequate resources and experienced practitioners can provide very-high-quality comprehensive pancreatic cancer care.

Pancreatic surgery in a community setting has some unique challenges. Pancreatic surgery requires an experienced surgeon and a skilled surgical assistant during much of the procedure. At academic and large community hospitals, surgical residents provide the skilled assistance necessary. Surgical residents from the University of Utah (BH and CJ) occasionally electively rotate at our hospitals and assisted on about 25 of these procedures. A certified nurse first assistant (JD) assisted on the majority of these procedures with other assistance from surgical partners where necessary. Additionally, in the absence of 24-hour surgical resident in-house availability, the 24-hour availability of hospitalists and intensivists at our facilities to provide critical care and urgent “rescue care” has been important.

Our relatively small numbers make any true statistical analysis impossible and irrelevant. However, our results can at least be assessed and understood in light of available national outcomes data and benchmarks. On this basis, our results are clearly acceptable when compared to large published series and databases. Our operative mortality was 2.9% for pancreatic procedures in general (2 of 70) and Whipple procedures specifically (1 of 34). This is consistent with other published series, even those with much higher volume. In the paper by Gordon et al. [2], in 1995, looking at volume of Whipple procedures and hospital mortality in Maryland (1988–93 data), the hospital with the highest volume had a mortality rate of 2.2%. Our series averaged 8 Whipples per year, which would be in the 4th of 5 quintiles described in that study. That group of hospitals had a mortality rate of 14.3%, compared to which, we are obviously very favorable. In a follow-up study of 1990–1995 Maryland State data which considered both Whipples and palliative procedures, Sosa et al. [3] stratified hospitals into low- (1–4), medium- (5–19), and high-volume (20 or more) procedures for pancreatic cancer. In our series, we averaged 16 pancreatic surgeries per year which would put us in the medium volume category. The mortality for pancreatic cancer resections and palliative bypasses at the medium volume hospitals in the Sosa study was 7% and 11%, respectively, compared to our mortality rate of 2% and 5% for the same categories. In the highest volume hospitals in the Sosa study, the respective mortality rates were 1% and 4%. In the Birkmeyer study which considered national Medicare data from 1994–1999 [5], the very highest volume hospitals (> than 16 pancreatic resections per year) had a mortality rate of 3.8%.

Another paper by Birkmeyer et al. [22] analyzed individual surgeon volume for a number of complex procedures including pancreatic resection. They analyzed Medicare claims data for 1998-1999 and stratified surgeons into low-, medium-, and high-volume groups. For pancreatic resection, 30-day mortality was 14.7%, 8.5%, and 4.6% for the three groups, respectively. The authors' definition of high volume in this paper was greater than 4 pancreatic resections per year. Thus, the surgeon in our paper would be stratified as a high-volume surgeon, and our 30-day operative mortality rate of 2.9% would be acceptable.

In terms of adjuvant therapy, our delivery of appropriate adjuvant chemo- and radiation therapy to 93% and 86% of eligible patients, respectively, is consistent with national standards. In fact, population-based studies by Davila et al. [23] and Birkmeyer et al. [6] of SEER-Medicare data from 1992–2002 demonstrated that only 49% of patients received adjuvant therapy after surgery. Differences between low- and high-volume centers were also seen, varying from 21% to 52% from low to high volume centers.

Nationally, participation in cancer clinical trials is 2–4% [24]. By comparison, we placed 14% of our patients into clinical trials in both adjuvant and palliative settings. Where patients are treated in local facilities with minimal travel burden, participation in clinical trials may well be more appealing to patients and families.

In our series, ten of 44 patients (23%) had unexpected metastatic disease at the time of exploration, and six patients (14%) had vascular involvement that precluded resection for a total unresectability rate of 36%. These results are comparable to typical published series including Massachusetts General Hospital [25], where the rate of unexpected metastatic disease was eight of 44 (18%) and 13 patients (30%) had vascular involvement for a total unresectability rate of 48% and University of Maryland [26] where the rate of unexpected metastatic disease was 27 of 101 (27%) and the rate of and 16 patients (16%) had vascular involvement for a total unresectability rate of 43%.

Overall survival, as well as survival by stage, remains difficult to compare because of our small numbers and 5-year survival is obviously unavailable as our experience only extends 4.5 years. Nevertheless, compared to NCDB data, our survival is clearly noninferior to these national data and may well be above average for one- and two-year survival. Our results are also comparable with typical large single institution series [27].

 The issue of the degree to which case volume influences outcomes in complex illnesses of all types and especially complex cancer surgery is important, and exhaustive data analyses have been done. The references [2–8] cited in this review are just a small sample of the available literature, but are specific for pancreatic cancer. These reviews and other large single-institution series have set clear outcome benchmarks that should be achievable by centers (large or small) seeking to provide comprehensive pancreatic cancer care. By every standard reported in this review—operative mortality, resectability rate, adjuvant chemo, and radiation therapy, participation in clinical trials, and overall survival, comparability to national tertiary care benchmarks has been achieved, even taking into consideration small numbers and a total experience of less than 5 years.

 There are a number of reasons that increasing the number of high-quality comprehensive community cancer facilities is desirable. Many patients, perhaps the majority, cannot or will not go to high-volume academic or regional cancer centers [7]. One of the implications of this is that a significant number of patients are not offered and do not receive potentially curative surgical therapy. This is well documented by Bilimoria et al. who used NCDB 1995–2004 data to demonstrate that 38% of potentially resectable patients were not offered surgery [28]. Tragically, their data also show that medically vulnerable patients—those over 65, black, Medicare or Medicaid, less educated, and less affluent–are overrepresented in this group. A second important issue is also demonstrated by Bilimoria's group. They used 1995–2005 NCDB data to demonstrate that wait times from diagnosis to treatment of many cancers, including pancreas, at NCI Comprehensive Cancer Centers and VA Hospitals increased during that decade [29]. They note that with the rising cancer burden in the US population this trend is likely to continue. Finally, data clearly show that patients prefer to be treated locally where possible. In fact many patients prefer to be treated locally even if the local facility has a demonstrably higher mortality rate than a regional center [10]. For all of these reasons, it is highly desirable that, where practical, community facilities and practitioners make the necessary investment to provide comprehensive complex cancer treatment.

## 10. Conclusions

This review documents the introduction of pancreatic surgery and with it, comprehensive multidisciplinary care of pancreatic cancer, to two community hospitals in whose geographic area such care was not previously available. Over a 4-year period, acceptable results, comparable to established tertiary care standards, for operative mortality, resectability rate, administration of adjuvant, and, where indicated, neoadjuvant therapy, clinical trial participation, and overall survival have been achieved.

Introduction of pancreatic surgery and providing multidisciplinary pancreatic cancer care is a resource-intense process. Medical institutions making that commitment must have full-time availability of biliary endoscopy, interventional radiology, surgical and anesthesia expertise in pancreaticobiliary procedures, intensive care medicine, as well as multidisciplinary oncologic treatment. Meeting the standards for Committee on Cancer Accreditation and implementing NCCN guidelines by a multidisciplinary team of practitioners and an active Cancer Committee are some of the important keys that can enable a community hospital to achieve excellence in pancreatic cancer care. Recognizing that judicious referral of extremely complex patients to regional centers is necessary is also important [30].

Many have advocated regionalizing complex cancer care in general and pancreatic cancer care specifically. The trend toward moving at least some complex pancreatic cancer away from low-volume centers is readily apparent [7]. Nevertheless, community-based cancer centers, hospitals, and physicians committed to providing complex cancer care can provide excellent care in that regard, despite being relatively low-volume centers. Such community centers are very beneficial to cancer patients in regards to the ability to offer more patients potentially curative therapies, reduce travel burden, and reduce wait times for therapy. Experience, commitment, and teamwork are as important as volume in the care of pancreatic cancer patients.

## Figures and Tables

**Table 1:** Survival in resected pancreatic cancer patients. A “potential” survivor is one whose surgery was done long enough ago to be a possible survivor for the indicated number of years.

	Total Patients	Potential 1-year survivors	Actual 1-year survivors	Potential 2-year survivors	Actual 2-year survivors	Potential 3-year survivors	Actual 3-year survivors
All resected patients*	28	21	18 (86%)	13	8 (62%)	8	2 (25%)
Stage I patients	8	7	7 (100%)	2	2 (100%)	1	1 (100%)
Stage II patients	19	13	10 (77%)	10	5 (50%)	7	1 (14%)

*Included in this group is the patient who presented with Stage III disease but was downstaged with neoadjuvant therapy and resected.
